# Recessive *PYROXD1* mutations cause adult-onset limb-girdle-type muscular dystrophy

**DOI:** 10.1007/s00415-018-9137-8

**Published:** 2018-12-04

**Authors:** Markus T. Sainio, Salla Välipakka, Bruno Rinaldi, Helena Lapatto, Anders Paetau, Simo Ojanen, Virginia Brilhante, Manu Jokela, Sanna Huovinen, Mari Auranen, Johanna Palmio, Sylvie Friant, Emil Ylikallio, Bjarne Udd, Henna Tyynismaa

**Affiliations:** 1 Research Programs Unit, Molecular Neurology, University of Helsinki, Helsinki, Finland; 2 Folkhälsan Institute of Genetics, Medicum, University of Helsinki, Helsinki, Finland; 3 Department of Molecular and Cellular Genetics, CNRS, GMGM-UMR7156, Université de Strasbourg, Strasbourg, France; 4 Department of Pathology, HUSLAB and University of Helsinki, Helsinki, Finland; 5 Division of Clinical Neurosciences, Turku University Hospital, University of Turku, Turku, Finland; 6 Department of Neurology, Neuromuscular Research Center, University Hospital and University of Tampere, Tampere, Finland; 7 Department of Pathology, Fimlab Laboratories, Tampere University Hospital, Tampere, Finland; 8 Clinical Neurosciences, Neurology, University of Helsinki and Helsinki University Hospital, Helsinki, Finland; 9 Neurology Department, Vasa Central Hospital, Vaasa, Finland; 10 Department of Clinical and Medical Genetics, University of Helsinki, Helsinki, Finland

**Keywords:** Limb-girdle muscular dystrophy, PYROXD1, Myopathy, Exome sequencing

## Abstract

**Objective:**

To describe adult-onset limb-girdle-type muscular dystrophy caused by biallelic variants in the *PYROXD1* gene, which has been recently linked to early-onset congenital myofibrillar myopathy.

**Methods:**

Whole exome sequencing was performed for adult-onset neuromuscular disease patients with no molecular diagnosis. Patients with *PYROXD1* variants underwent clinical characterization, lower limb muscle MRI, muscle biopsy and spirometry. A yeast complementation assay was used to determine the biochemical consequences of the genetic variants.

**Results:**

We identified four patients with biallelic *PYROXD1* variants. Three patients, who had symptom onset in their 20s or 30s, were homozygous for the previously described p.Asn155Ser. The fourth patient, with symptom onset at age 49, was compound heterozygous for p.Asn155Ser variant and previously unknown p.Tyr354Cys. All patients presented with a LGMD-type phenotype of symmetric muscle weakness and wasting. Symptoms started in proximal muscles of the lower limbs, and progressed slowly to involve also upper limbs in a proximal-predominant fashion. All patients remained ambulant past the age of 60. They had restrictive lung disease but no cardiac impairment. Muscle MRI showed strong involvement of anterolateral thigh muscles. Muscle biopsy displayed chronic myopathic changes. Yeast complementation assay demonstrated the p.Tyr354Cys mutation to impair PYROXD1 oxidoreductase ability.

**Conclusion:**

*PYROXD1* variants can cause an adult-onset slowly progressive LGMD-type phenotype.

**Electronic supplementary material:**

The online version of this article (10.1007/s00415-018-9137-8) contains supplementary material, which is available to authorized users.

## Introduction

Inherited disorders of the skeletal muscles are classified based on clinical, histopathological and genetic features. Limb-girdle muscular dystrophy (LGMD) is a genetically heterogeneous group of muscular dystrophies with autosomal inheritance characterized by slowly progressive degeneration of proximal limb muscles. In many types of LGMD also other muscles, e.g., distal muscles, respiratory muscle and the heart may be affected. Inheritance can be either dominant (LGMD1) or recessive (LGMD2), and to date at least 34 LGMD disease genes are known [1–3]. Distribution of muscle involvement, age at onset and rate of progression show great variability, which are underscored by the extensive genetic diversity.

Biallelic *PYROXD1* gene mutations were identified to underlie congenital myopathy in nine patients from five different families [4]. These patients had the onset of muscle weakness between birth and 8 years with slow disease progression, and muscle pathology consistent with myofibrillar myopathy. All patients were still ambulant at the time of study, the oldest patient being 31 years of age. *PYROXD1* encodes a nuclear-cytoplasmic oxidoreductase, with a currently unknown exact function in cellular redox regulation [4]. Patients from four families had a mutation causing p.Asn155Ser amino acid change, which was shown to impair reductase activity in a complementation assay of yeast lacking glutathione reductase [4]. Another patient with homozygous p.Asn155Ser variant was recently reported, presenting with progressive muscle weakness starting at the age of 9 years and leading to loss of ambulation at the age of 37 years [5].

Here we describe additional four patients from three families with recessive *PYROXD1* variants. In contrast to the previous report, our patients had the disease onset in adulthood and have now reached 60 years of age, allowing evaluation of the natural history of *PYROXD1* associated disease.

## Materials and methods

### Patients

**Fig. 1:**
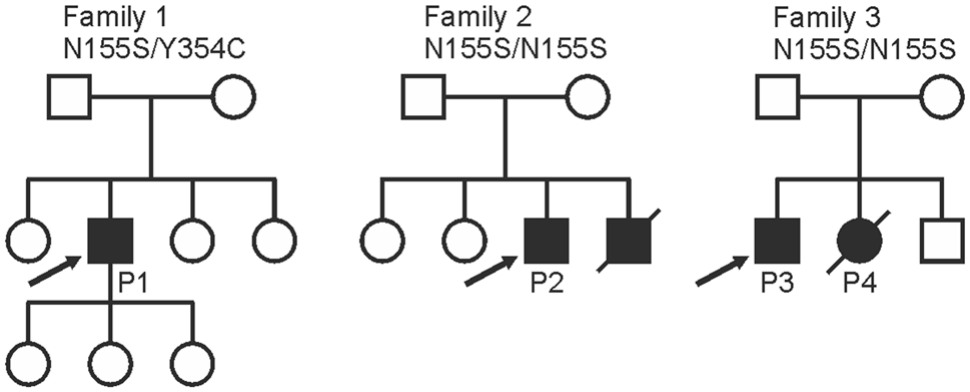
Family pedigrees and the identified *PYROXD1* variants

The patients in this study are from three families of Finnish origin with non-consanguineous parents (Fig.1). Patients; patient 1 (P1, Family 1), patient 2 (P2, Family 2), and siblings patient 3 and 4 (P3 and P4, Family 3), were studied in cohorts of undiagnosed neuromuscular disease patients. In addition to detailed clinical neurological examinations, all patients underwent electroneuromyography (ENMG) investigations, muscle biopsy, lower limb muscle MRI, spirometry/respiratory assessment and measurement of serum creatine kinase (CK) values (Table1). The MR images of patient P4 had been performed a long time ago at age 45 years and therefore only the written radiology report was available for review. Whole-body MRI had been performed in two patients (P1 and P2).

**Table 1:** Clinical features of the patients

Family	1	2	3	3
Patient	P1	P2	P3	P4
Gender, age	Male, 65 years	Male, 65 years	Male, 70 years	Female, 70 years^a^
Ethnicity, consanguinity	Finnish, no	Finnish, no	Finnish, no	Finnish, no
PYROXD1 variants	c.464A > G (p.Asn155Ser, chr12: g.21605064A > G), c.1061A > G (p. Tyr354Cys, chr12:g. 12: 21615741A > G)	Hom, c.464A > G (p.Asn155Ser, chr12: g.21605064A > G)	Hom, c.464A > G (p.Asn155Ser, chr12: g.21605064A > G)	Hom, c.464A > G (p.Asn155Ser, chr12: g.21605064A > G)
Onset/progression	49 years, slowly progressive	10 years, slowly progressive	30 years, slowly progressive	33 years, slowly progressive
Age on last comprehensive examination, severity	63 years, ambulant without aids, help from both hands when rising from chair	64 years, ambulant with two sticks, kyphotic posture and atrophic upper back muscles	70 years, ambulant with 1–2 sticks for 200 m, severe proximal upper and lower limb weakness	70 years, wheelchair bound (66 years)
Restrictive lung disease	Yes, FVC 54% (63 years)	Yes, episodic dyspnea and FVC 40% (64 years)	Yes, FVC 67% with severely reduced MIP, MEP and PCF values (70 years)	Yes, FVC 42% (59 years) and 30% (68 years)
Distal and proximal upper limb strengths	Prox. 4/5 Dist. 5/5	Prox. 2/5 Dist. 4/5	Prox. 3/5, 2/5 Dist. 4/5	Prox. 4/5, 2/5 Dist. 3.5/5
Hip flexion strength	3/5	2/5	1/5	2/5
Knee flexion/extension	Flex. 4/5 Ext. 4/5	Flex. 3/5 Ext. 2/5	Flex. 3/5 Ext. 2/5	Ext. 2/5
Distal lower limb strengths	Ankle plantar and dorsal flexion 4/5 (64 years)	Ankle dorsal flexion 3/5, plantar flexion 5/5 (65 years)	Ankle plantar and dorsal flexion 3/5 (70 years)	Ankle plantar and dorsal flexion 5/5 (45 years)
Spinal and truncal muscles	Moderate volume loss	Atrophic upper back muscles	Neck flexor, spinal and abdominal muscle weakness	Neck flexor and abdominal muscle weakness
EMG	Proximal and distal muscle abnormal motor unit potentials	Myopathic changes	Myopathic changes in proximal and paraspinal muscles	Myopathic changes in proximal muscles
Neurography	Normal	n.a	Normal	Normal
MRI	Symmetric atrophy and fatty replacement in all lower limb muscles, mild proximal atrophy in upper limbs and significant atrophy in muscles of the pectoral girdle	Symmetric atrophy and fat replacement in all muscle compartments. In the lower limbs, atrophy was found rather uniformly, whereas in the upper limbs it was relatively more advanced in dorsal compared to ventral muscles (61 years)	Wide spread fatty replacement in gluteal, thigh and lower leg muscles (65 years)	Most severe fatty replacement in semitendinosus, sartorius, gracilis. and gastrocnemius medialis muscles (45 years)
Biopsy	Dystrophic	Dystrophic	Dystrophic	Dystrophic
CK	Normal	288–340 U/l	Normal	Normal
Acylcarnitine profile	Normal	n.a	n.a	n.a
Cardiac ultrasound	Normal	Normal (61 years)	Normal (66 years)	n.a

*FVC* forced vital capacity, *MIP* maximum inspiratory pressure, *MEP* maximum expiratory pressure, *PCF* peak cough flow

^a^Age at death due to respiratory insufficiency and pneumonia

Muscle biopsies were histochemically stained with haematoxylin & eosin (H&E), Gomöri trichrome, nicotinamide adenine dinucleotide tetrazolium reductase (NADH-TR) and combined cytochrome oxidase (COX)/succinate dehydrogenase (SDH). The following immunohistochemical stainings were performed for P1 and P3: MyHC double staining [Myosin Heavy Chain, Slow, Myosin Heavy Chain, A4.74 (fast)], p62, myotilin and desmin. Furthermore, biopsies from P1 and P2 were stained for sarcolemmal proteins dystrophin 1–3, dysferlin, sarcoglycan-alpha, dystroglycan-alpha and caveolin-3 in addition to merosin and emerin. For P2, only archival histological and histochemical stainings were available, and thus MyHC, p62, myotilin or desmin immunohistochemical stainings were not possible.

All subjects in this study have provided written consent for the use of clinical data and material. The study was approved by Helsinki University Hospital and Tampere University Hospital ethics boards.

### DNA sequencing

For P1 and P2, whole exome sequencing (WES) was performed at the Finnish Institute of Molecular Medicine (FIMM). Briefly, 150ng of gDNA was fragmented with a Covaris E220 evolution instrument (Covaris). Sample libraries were processed according to SeqCapEZ Library SR (Roche Nimblegen) manual. NimbleGen capture was performed according to NimbleGen SeqCap EZ Exome Library SR User’s Guide. Sequencing was performed with Illumina HiSeq2500 system in Rapid mode using HiSeq Rapid v2 kits (Illumina). Reads were then aligned to the GRCh37 reference genome with the BWA (0.6.2), and the mpileup from the SAMTOOLS (1.4) package was used for variant calling.

For family 3 (P3, P4 and their unaffected brother), WES was performed essentially by the same method, but library preparation was done using KAPA Hyper library preparation Kit (Kapa Biosystems, Wilmington, Ma, USA) and SeqCap EZ MedExome assay (Roche Nimblegen) was used for target enrichment.

Sanger sequencing was performed with primers specific for *PYROXD1* exon 5 (CAGTGGGAAAGTGAGATTCATTT and ATTACGGATTCCACAAGAGCT) and exon 10 (CCATGGAAATTCAGCTCAGGT and AACAACTGTGCTAGCTTCCT).

### Plasmids, strains, media, and methods for yeast cells

The human *PYROXD1* and *PYROXD1*-p.Tyr354Cys cDNAs were cloned by the Gateway® (Invitrogen) method into pDONR221 entry vector and then recombined into yeast destination vectors (Addgene;[6]) to obtain pAG415-promGPD-PYROXD1 (pSF371) and pAG415-promGPD-PYROXD1-p.Tyr354Cys (pSF501) plasmids. The pAG415 is a low-copy number CEN plasmid bearing the constitutive GPD (glyceraldehyde-3-phosphate dehydrogenase) promoter and the *LEU2* auxotrophic marker for selection of the transformants on SC-Leu medium. Plasmid sequences were verified (GATC Biotech). The *Saccharomyces cerevisiae* wild-type BY4742 (*MATα leu2*Δ*0 ura3*Δ*0 his3*Δ*0 lys2*Δ*0*) reference strain and the *glr1∆* (*MATα leu2*Δ*0 ura3*Δ*0 his3*Δ*0 lys2*Δ*0 glr1::KanMX*) mutant strain were used. Yeast cells were transformed using the modified lithium acetate method [7]. The indicated yeast strains were grown at 30°C to mid-exponential growth phase in synthetic complete (SC) medium SC-Leu: 0.67% yeast nitrogen base (YNB) without amino acids, 2% glucose and the appropriate—Leu dropout mix, to maintain the plasmid. These precultures were used to inoculate the rich medium YPD: 1% yeast extract, 2% peptone, 2% glucose, or the oxidative stress medium YPD + H_2_O_2_: 1% yeast extract, 2% peptone, 2% glucose, 3mM H_2_O_2_, and the growth of the yeast cells was analysed at 30°C under agitation in liquid medium by measuring the OD at 600nm over time, with measurements every 10min over 17h.

For western blot analysis, total yeast protein extracts were prepared by NaOH lysis of 1.5 OD_600nm_ unit of yeast cells, followed by trichloroacetic acid (TCA) precipitation and the pellet was resuspended in 50µl of 2X Laemmli buffer plus Tris Base. Samples were incubated 5 min at 37°C prior western-blot analysis by 8% SDS-PAGE, followed by transfer on a nitrocellulose blotting membrane (Amersham™ Protran™ 0.45µm NC) and immunoblotting with rabbit polyclonal anti-PYROXD1 (1/500, R3500) antibodies [4] using standard procedures. Images were acquired with the ChemiDoc Touch Imaging System (Bio-Rad).

## Results

### Clinical features of P1

Patient 1 (Family 1) is a male who had been previously diagnosed with Ménière’s disease, but had been otherwise healthy. He had slowly progressive proximal muscle weakness combined with back pain starting at the age of 49 years (Table1). Before this his strengths had been normal, and he reported no problems with doing sports as a child or young adult. A lumbar disk herniation had been operated at the age of 51 years. At the age of 60 years, he was referred for neurologic consultation because of an incidental MRI finding of atrophy and fat replacement symmetrically in the paravertebral lower back muscles. The family history was negative for neuromuscular disease.

On clinical examination at the age of 63 years, he was ambulant without aids, but needed to use both hands when rising from a chair or climbing stairs. Moderate volume loss was noted in the upper paraspinal muscles, but otherwise there was no evident atrophy. Distally upper limb strengths were normal, proximally slightly reduced on both sides. In the lower limbs, hip flexion and knee flexion/extension strengths were decreased on both sides. Distal lower limb strengths were within normal limits. There was no facial or bulbar weakness.

Electromyography (EMG) of both proximal and distal muscles in all limbs showed abnormal motor unit potentials (MUPs), either small polyphasic or large and partially polyphasic. Nerve conduction studies showed normal conduction velocities and sensory and motor amplitudes.

Plasma creatine kinase (CK), acylcarnitine profile and cardiac ultrasound were normal. Spirometry showed evidence of moderate restrictive lung disease. High resolution CT of the thorax showed nodular parenchymal change consistent with pulmonary sarcoidosis. He was placed on an inhaled glucocorticoid.

### Clinical features of P2

In his late teens before the age of 20 years, patient 2 (Family 2) needed support from his hands to climb stairs and he had trouble running. Nevertheless, he played football at the age of 35 years, and started requiring walking aids only at the age of 54 years. On examination at the age of 64 years he walked with two sticks, and short distances without aids. He had kyphotic posture and upper back muscles were noted as atrophic.

There was slight ptosis and facial weakness bilaterally. Limb strength was decreased predominantly proximally in a symmetric fashion. Arm elevation was less than 90° and elbow extension was severely decreased. Distal hand muscle strength was better preserved. In the lower limbs, hip flexion, knee extension and ankle dorsiflexion/plantar flexion were severely affected, while knee flexion was moderately reduced.

EMG showed myopathic changes. Plasma CK was slightly increased. Cardiac ultrasound at age 61 years was normal. He had episodic dyspnea and reduced respiratory function (age 64 years), but so far has not required ventilator support.

The patient’s brother had also been diagnosed with muscle disease and died of pneumonia at the age of 43 years. Both parents and two older sisters were healthy.

### Clinical features of patients P3 and P4

The proband P3 and his sister P4 (Family 3) were asymptomatic until early thirties when they noticed weakness in their proximal lower limbs. They had difficulties rising from squat or chairs. Few years later muscle weakness was also reported in proximal upper limbs. The progression was very slow. At the age of 70 years the proband was ambulant with sticks for 200 m but rising from low chair was impossible. Gait was waddling with hyperextended knees. He could elevate his arms less than 90°. His posture was normal although spinal and abdominal muscles were weak with mild neck flexor weakness. Also distal lower limb muscles were weak (ankle plantar and dorsal flexion grade 3). Scapular winging or facial weakness was not observed. He had hoarseness in his voice but no other bulbar symptoms. He had had two strokes (left hemisphere aged 61 years and right hemisphere 66 years), which might contribute to the symptoms although acute hemiplegic symptoms had resolved. There was severe symmetrical muscle atrophy in the anterior part of the thigh, proximal upper limbs and shoulder girdle. He underwent echocardiography with normal results. Mild respiratory insufficiency and weak cough strength was observed in pulmonary function tests.

The sister, P4, started to use rollator at the age of 65 years and wheelchair aged 66 years. She could not extend her knees and hip flexion was weak as well as neck flexors and abdominal muscles. She had asymmetrical upper limb weakness both proximally and distally with left side more severely affected. No bulbar symptoms were present. She had severe progressive respiratory insufficiency and she died from pneumonia aged 70 years. There were no cardiac symptoms; echocardiography was not performed. Both siblings had normal CK levels. EMG showed myopathic changes especially in the proximal upper and lower limbs.

### Muscle MRI and biopsy

**Fig. 2:**
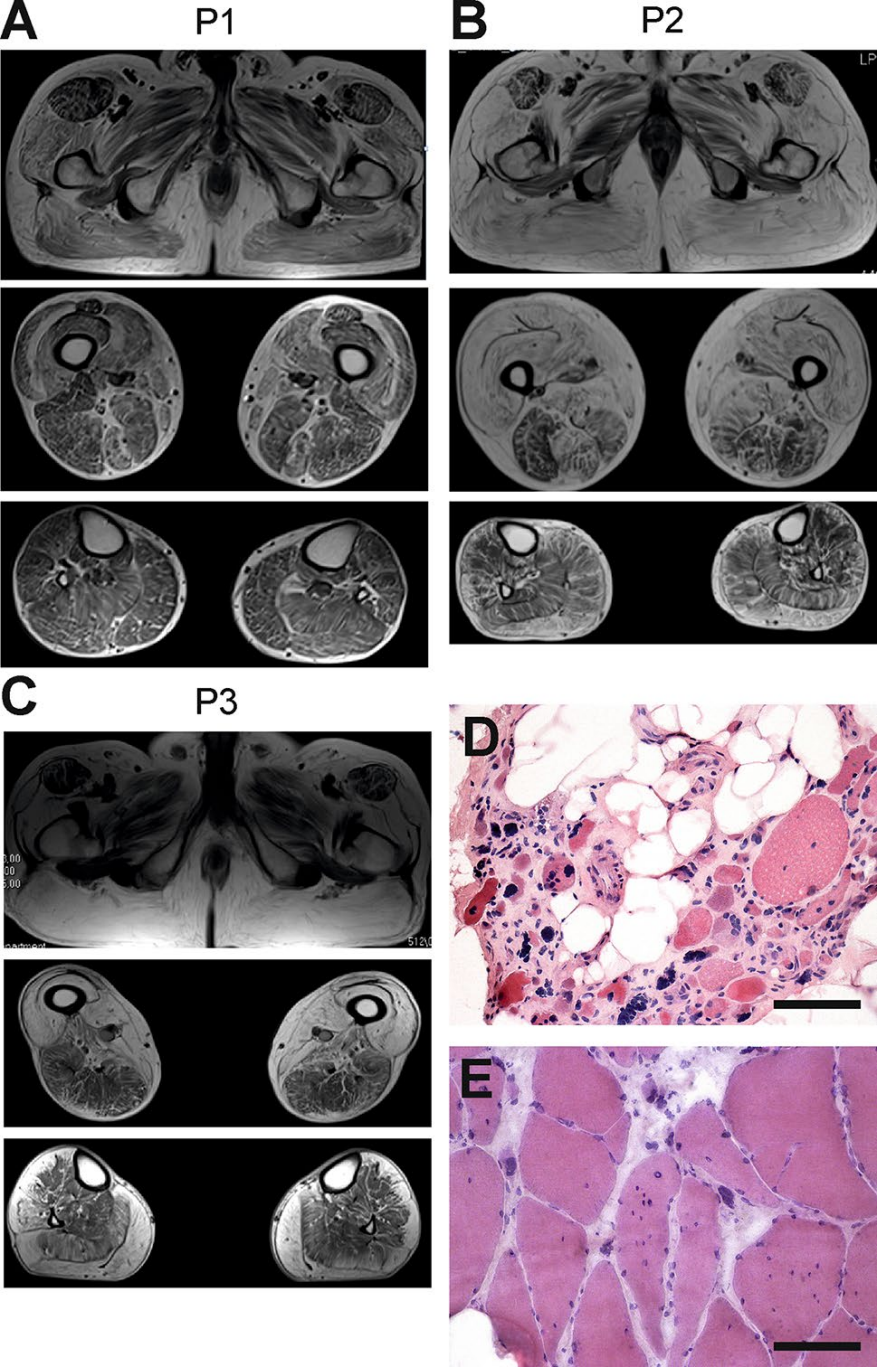
Muscle MRI and biopsies. MRI was performed for **a** individual P1 at age 60, **b** individual P2 at age 59, and **c** individual P3 at age 65. Gluteus maximus, quadriceps, sartorius and gracilis muscles were most severely affected in all patients, although the most proximal parts of the rectus femoris muscles were relatively spared or even hypertrophic. Anterior thigh muscles were always more severely affected than the hamstrings and adductor compartments. A very peculiar pattern of fatty degeneration was observed in patients P2 and P3, where only the outer parts of the anterior and lateral compartment muscles were replaced by fat. In patient P1, there was an unusual crescent-shaped fatty-degeneration involving the inner parts of vastus lateralis and medialis muscles. Muscle biopsy (vastus lateralis) from **d** individual P2 and **e** individual P1 showed a dystrophic pattern, where individual P2 had an almost end-stage pathology with severe atrophy, fibrosis and fatty infiltration. Scale bars 100µm

Muscle MRI in all patients (P1, P2, P3 and P4) showed an unusual pattern of fatty dystrophic changes with total or near-total replacement of gluteus maximus, sartorius, gracilis and quadriceps (Fig.2a–c). Similar to the original report on *PYROXD1*-related myopathy [4], rectus femoris was less affected than the vasti muscles. In the distal lower limbs, gastrocnemii were the most affected muscles in patients P2, P3 and P4, while all lower leg muscles were only mildly and diffusely affected in patient P1. In two patients (P2 and P3), a very peculiar pattern of fatty degeneration was observed, where the outer parts of the anterior and lateral compartment muscles were replaced by fat, but not the inner portions (Fig.2b, c). No significant STIR edema was detected in any muscle, which is consistent with a relatively inactive and chronic degenerative process. Muscles of the upper girdle and the torso displayed diffuse fatty degenerative changes to a mild-moderate degree, while the muscles of the forearm and hand were minimally affected or entirely spared (not shown).

Muscle biopsies from the vastus lateralis of P2 (Fig.2d) and P1 (Fig.2e) showed chronic myopathic changes with atrophy, fat infiltration and strong fiber size variability, multiple internalized nuclei, and without significant myofibrillar pathology. Immunohistochemistry of sarcolemmal membrane-associated proteins, merosin and emerin was normal. Stainings for p62, myotilin and desmin in P1 showed no myofibrillar features.

The first biopsies of the siblings, P3 and P4, were performed 30 years ago and were not available for further analysis. The morphological changes were reported to be dystrophic without specific features. Patient 3 underwent a new biopsy from tibialis anterior muscle at the age of 70. The muscle pathology showed extensive end-stage dystrophic changes. No fiber type predominance was noted. Immunohistochemical staining for myotilin revealed a few positive cytoplasmic inclusions in scattered atrophic muscle fibers (Supplementary Fig.1). However, no desmin- or p62-positive protein aggregates were found.

### Genetic findings

The patients’ pedigrees suggested recessively inherited disease (Fig.1). Thus, the exome sequencing data were filtered for homozygous or compound heterozygous variants that induce damaging changes to amino acid sequence, have population frequency of less than 0.001 in the total population and Finnish sub-population in ExAC variant database, CADD-scores of 20 or greater, and are present in less than 1% of an in-house database with 429 samples (P1 and P2) or less than 4% of a separate in-house database of 63 samples (P3 and P4). For P2, P3 and P4, the homozygous variant c.464A > G p.Asn155Ser in *PYROXD1* (ENST00000240651.9) caught our attention because of its recent association with myopathy [4]. In the previous study, this variant was identified in four out of five studied families as homozygous (two families) or compound heterozygous (two families), and it is present in variant databases as a low frequency European variant always in heterozygous state. According to the GnomAD database, its allele frequency is slightly higher in the Finnish population (1.186 × 10^−4^) than elsewhere. The unaffected brother in the family 3 was a heterozygous carrier of the p.Asn155Ser variant.

**Fig. 3:**
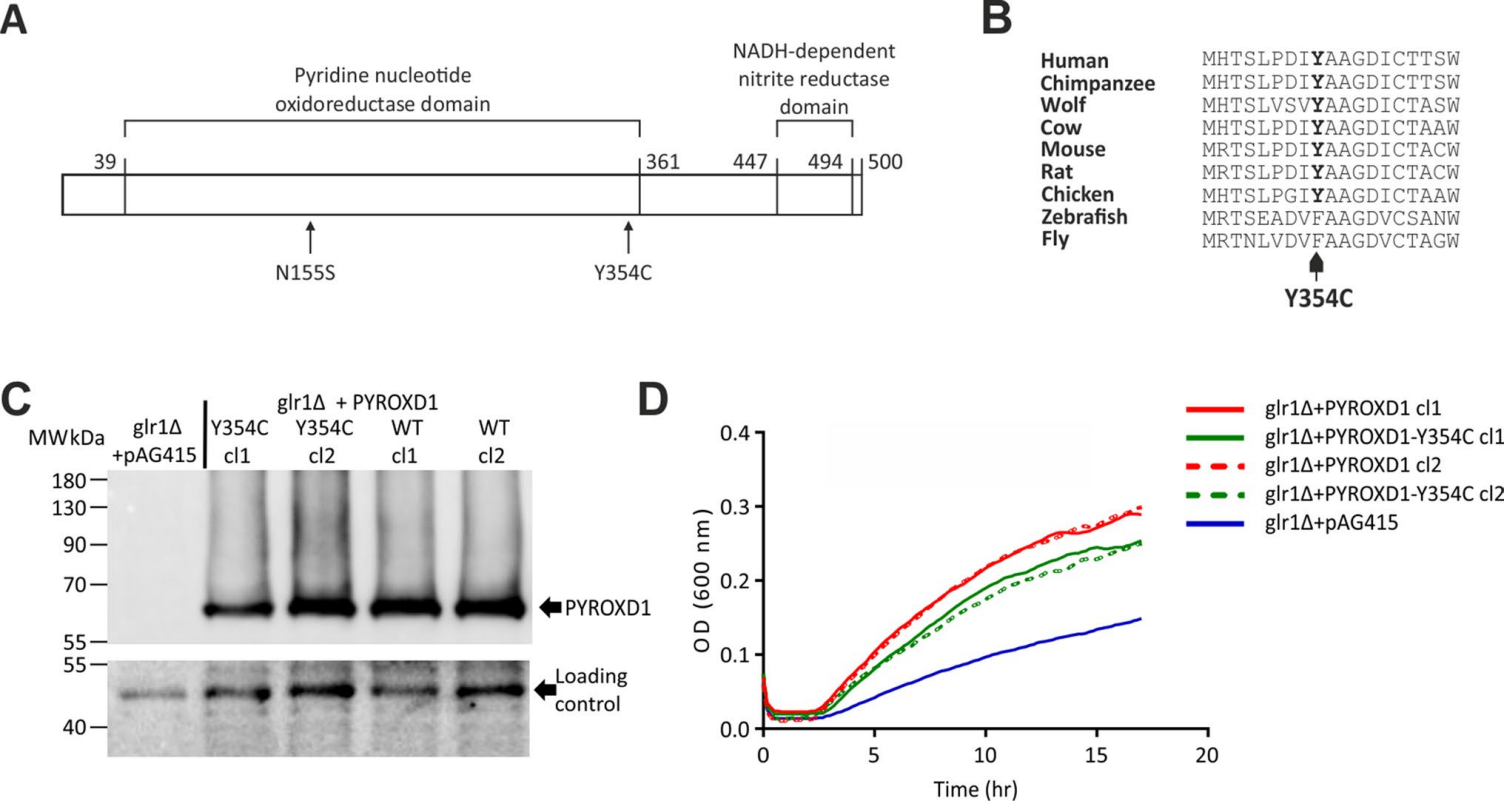
PYROXD1 variants identified in this study and the functional characterization of p.Tyr354Cys in a yeast complementation assay. **a** A schematic of PYROXD1 with functional domains and the missense variants of patients of this study. **b** Evolutionary conservation of Tyr354. **c** Western blot of *glr1*Δ yeast cells transformed with yeast expression vectors empty (pAG415) or bearing the PYROXD1 wild-type (WT) or p.Tyr354Cys (Y354C) under the control of a constitutive promoter, two different clones (cl1 and cl2) were analyzed. The black arrows indicate the PYROXD1 protein and the loading control. **d** The indicated yeast cells were grown at 30 °C in rich medium (YPD) containing H_2_O_2_ (3 mM) to induce an oxidative stress. The growth curves of the different strains were determined by measuring the cell growth (OD at 600 nm) over time (h)

Patient 1 was compound heterozygous for the p.Asn155Ser and a previously unknown variant c.1061A > G p.Tyr354Cys. The latter variant is found in four heterozygous individuals in the GnomAD database with a frequency of 1.444 × 10^−5^. The mutated tyrosine is located in the N-terminal end of the pyridine nucleotide oxidoreductase domain of PYROXD1 (Fig.3a), and the amino acid is evolutionarily conserved (Fig.3b). Segregation of the variants was investigated in the family of patient 1, and the affected individual was the only one carrying both mutations. The p.Asn155Ser allele was inherited maternally and the p.Tyr354Cys paternally.

### The PYROXD1-p.Tyr354Cys is defective in oxidative stress resistance in yeast cells

Humanization of *Saccharomyces cerevisiae* yeast cells can be used to better understand and/or to identify the cellular role of a human protein [8, 9]. Indeed, the human *PYROXD1* cDNA was shown to rescue the oxidative stress defect associated with the yeast glutathione oxidoreductase *glr1∆* deletion mutant strain [4]. Furthermore, the p.Asn155Ser variant was shown to have impaired rescue ability in the yeast complementation assay [4]. We tested here the effect of the new p.Tyr354Cys variant using the same yeast *glr1∆* strain. The *glr1∆* mutant cells were transformed by empty plasmid (pAG415) or by plasmids bearing either *PYROXD1* wild-type cDNA, or the mutant allele p.Tyr354Cys. Expression of the human cDNAs was controlled by western blot analysis with anti-PYROXD1 antibody, showing that the wild-type and the mutant form of PYROXD1 were expressed in two different clones (cl1 and cl2) of yeast cells (Fig.3c).

We observed that the yeast glutathione reductase *glr1∆* mutant had a strong growth defect when grown in the presence of hydrogen peroxide (Fig.3d). As shown before, the expression of the human *PYROXD1* cDNA was able to complement the growth defect of the *glr1∆* mutant cells [4], indicating that PYROXD1 has an oxidoreductase activity in vivo in yeast cells. Compared to wild-type PYROXD1, the p.Tyr354Cys variant had a lower growth rate in the presence of H_2_O_2_, showing that this new patient mutation impairs the oxidoreductase activity of PYROXD1 in vivo in yeast cells (Fig.3d).

## Discussion

We report here new patients with recessive *PYROXD1* variants underlying myopathy phenotypes. In contrast to the congenital myopathy cases described previously [4, 5], our patients had a considerably later disease onset and lacked significant myofibrillar pathology. In fact, a few myotilin-positive inclusions were only observed in the end-stage degenerated muscle of one of our patients. The clinical picture of our patients was consistent with adult-onset, slowly progressive LGMD type disease. Reasons for the clear difference in disease onset between the patients of Turkish [4] and Sudanese of Arab [5] ancestry, and the Finnish patients of this study, who were homozygous for the p.Asn155Ser variant, are speculative at this point but may involve the population-specific genetic background or some environmental factors that are relevant for the regulation of PYROXD1 oxidoreductase activity.

Muscle MRI may help to distinguish *PYROXD1* myopathy from other myopathies, as our patients showed an unusual preference for anterolateral thigh muscles. Overall, the clinical course of the disease appears relatively benign, with preservation of ambulation past the age of 60. Respiratory impairment is a concern, however, as all our patients had developed respiratory muscle weakness and the brother of P2, who had been affected by a very similar kind of progressive muscle weakness, died from a respiratory infection at age 43 years. Also a 21-year-old patient described by O’Grady et al. had restrictive lung disease [4]. Regular monitoring of pulmonary function is therefore mandated in *PYROXD1* myopathy. Fortunately, there appears to be no cardiac involvement.

It is important to note that the aged individuals in this study did not have evidence of neuropathy. While two affected individuals aged 26 and 29 years described by O’Grady et al. had an axonal neuropathy [4], our finding suggests that neuropathy is not a universal consequence of *PYROXD1* variants even with advancing age. An important feature to note is axial myopathy, which was prominent in P1, and also present in 8/9 of O’Grady’s patients. It may be of interest to look for *PYROXD1* variants in individuals presenting with pure axial myopathy, the genetics of which remains incompletely studied [10].

Three of our four patients were homozygous for the previously described p.Asn155Ser, whereas one was compound heterozygous for the same variant and the previously undescribed p.Tyr354Cys. Both of these variants are found in different populations according to exome and genome databases, suggesting that additional patients will probably be identified. The p.Asn155Ser change appears to be a common pathogenic *PYROXD1* variant, as most described patients carry it in one or both alleles. However, the associated myopathy can have highly variable ages of onset. Our patient, P1, who was compound heterozygous for the p.Tyr354Cys variant together with p.Asn155Ser had the highest age of onset of all patients, suggesting that p.Tyr354Cys might impair the function of PYROXD1 less than the p.Asn155Ser variant.

In conclusion, our study shows that *PYROXD1* should be considered as a causative gene also in later-onset unsolved myopathies, which resemble LGMD.

## Electronic supplementary material

Below is the link to the electronic supplementary material.

Supplementary material 1 (PDF 238 KB)
