# Supplementary material for: Recessive PYROXD1 mutations cause adult-onset limb-girdle-type muscular dystrophy
Source: J Neurol. 2018 Dec 4;266(2):353–60. doi: 10.1007/s00415-018-9137-8 (PMC6373352; doi:10.1007/s00415-018-9137-8)
Supplement: Supplementary file 1 — Supplementary material 1 (PDF 238 KB) [file 415_2018_9137_MOESM1_ESM.pdf]

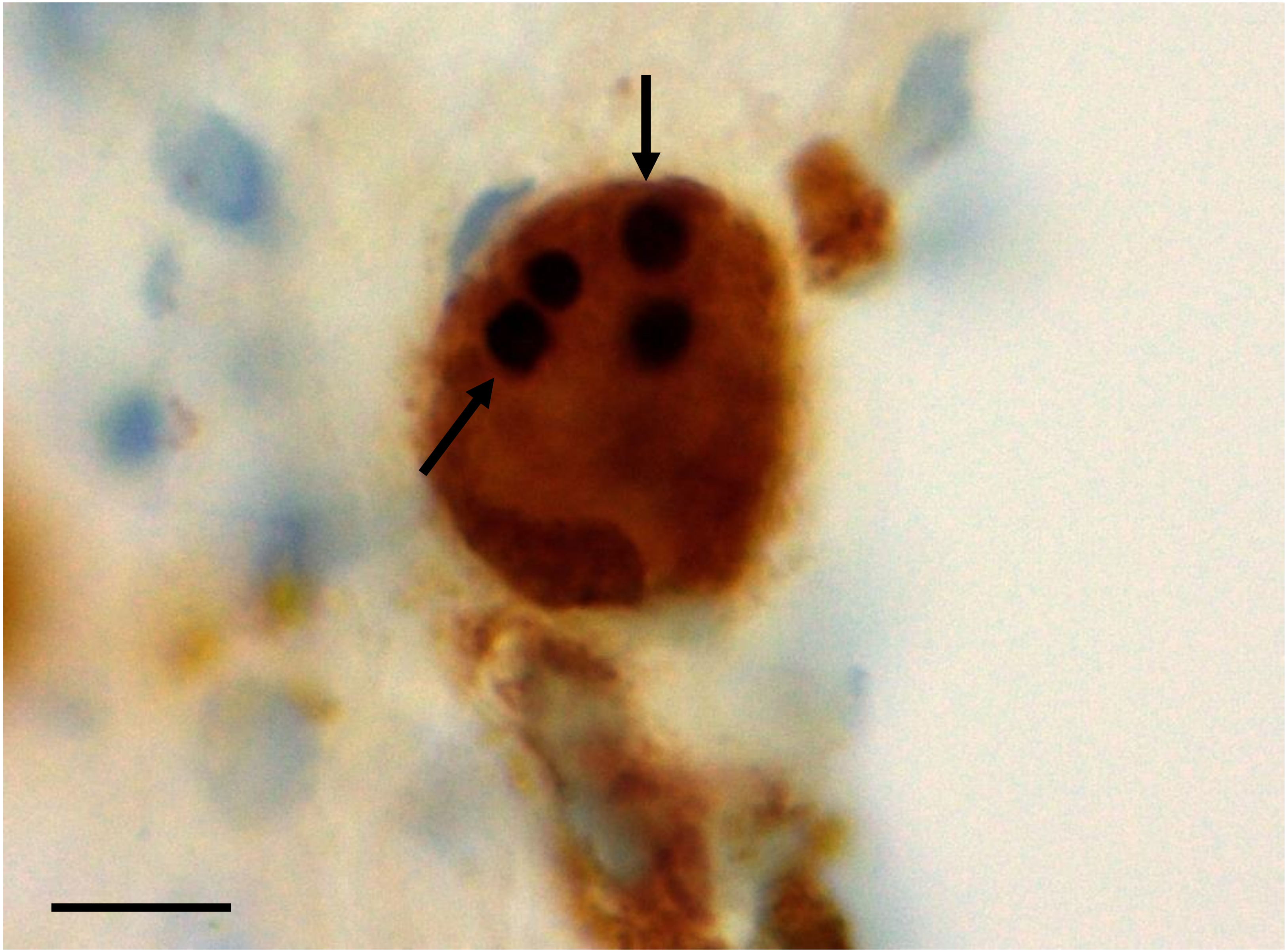

Supplementary figure 1. Myotilin immunostaining in P3 shows four intensively positive rounded cytoplasmic inclusion bodies (arrows) in a highly atrophic muscle fiber. Scale bar = 10 µm.
